# AP-1 signaling pathway promotes pro-IL-1β transcription to facilitate NLRP3 inflammasome activation upon influenza A virus infection

**DOI:** 10.1080/21505594.2022.2040188

**Published:** 2022-03-17

**Authors:** Pin Wan, Simeng Zhang, Zhihui Ruan, Xueli Liu, Ge Yang, Yaling Jia, Yongkui Li, Pan Pan, Wenbiao Wang, Geng Li, Xulin Chen, Zhixin Liu, Qiwei Zhang, Zhen Luo, Jianguo Wu

**Affiliations:** a Foshan Institute of Medical Microbiology, Foshan, China; b Guangdong Provincial Key Laboratory of Virology, Institute of Medical Microbiology, Jinan University, Guangzhou, China; c State Key Laboratory of Virology, College of Life Sciences, Wuhan University, Wuhan, China; d The First Affiliated Hospital of Jinan University, Jinan University, Guangzhou, China; e Department of Infectious Diseases, Department of Respiratory, Renmin Hospital, School of Basic Medical Sciences, Hubei University of Medicine, Shiyan, China

**Keywords:** AP-1 signaling pathway, influenza A virus, IAV, interleukin-1β, IL-1β, NLRP3 inflammasome, type I interferon

## Abstract

NLRP3 inflammasome mainly controls interleukin-1β (IL-1β) secretion, leading to cell death called pyroptosis constituting a major antiviral host defense and inflammatory diseases upon viral infection. The RAF-MEK1/2-ERK1/2 cascade and downstream c-Jun/Fos and Activator protein-1 (AP1) signaling pathway control the degree of inflammatory response. Influenza A virus (IAV) infection is known to stimulate NLRP3 inflammasome activation and inflammatory responses. Nevertheless, the detailed mechanism by which IAV induces NLRP3 inflammasome activation involved in transcription of pro-IL-1β mRNA remains elusive. In our study, we found that IAV infection promotes pro-IL-1β mRNA transcription and activates NLRP3 inflammasome. Detailed studies reveal that type I interferon (IFN-α/IFN-β) as well as U0126 (a selective inhibitor of MEK-1 and MEK-2) typically inhibit IAV-mediated NLRP3 inflammasome activation via downregulating pro-IL-1β mRNA. Moreover, knock-down of c-Jun decreases pro-IL-1β mRNA and inhibits NLRP3 inflammasome activation upon IAV infection. Overall, the findings uncover that AP-1 signaling pathway promotes NLRP3 inflammasome activation upon IAV infection, which provides a new idea for the therapy of NLRP3 inflammasome-associated inflammatory diseases.

## Introduction

NOD-like receptors (NLRs), belonging to the pattern recognition receptors (PRRs) family members, play important roles in innate immunity [1–3]. NLR family pyrin domain containing protein 3 (NLRP3) is the most extensively concerned receptor in NLRs [4]. It is capable of being stimulated by pathogen or damage-associated molecular patterns (PAMPs or DAMPs), including virus, bacteria, fungi, ATP, nigericin, MSU, silica, and aluminum [5–10].

Upon recognizing PAMPs or DAMPs, NLRP3 protein starts oligomerization, subsequently binds ASC and Caspase-1 to assemble into NLRP3 inflammasome platforms, finally facilitating maturation of pro-IL-1β and cell death called pyroptosis [3,11]. Activation of canonical NLRP3 inflammasome needs two steps: the first step is that PAMPs activate NF-κB to accelerate both NLRP3 and pro-IL-1β mRNAs, thereby controlling threshold of NLRP3 inflammasome; the second step is that PAMPs or DAMPs activate the NLRP3 inflammasome [12,13]. NLRP3 inflammasome is critical to regulate immune responses against many kinds of viruses, including IAV [13–15].

IAV infection may cause a highly pathogenic and infectious disease of human and many other animals (such as pigs and chickens). The highly pathogenic IAV caused pandemics, leading to high mortality, such as Spain flu in 1918 and Hong Kong flu in 1997 [16–18]. During this process, the tissue damages caused by NLRP3-mediated excessive pro-inflammatory responses in host immune cells was considered as the main factor contributing to high mortality upon IAV infection [19,20]. It is now known that NLRP3 inflammasome is closely involved in IAV-mediated immune responses [21–26].

Emerging evidence provided that IAV infection activates the NLRP3 inflammasome by multiple ways. IAV RNA stimulates NLRP3 inflammasome through lysosomal maturation or reactive oxygen species [21]. M2 protein of IAV activates NLRP3 inflammasome through its intracellular M2 ion channel [23], while IAV virulence protein PB1-F2 can activate NLRP3 inflammasome [24]. Conversely, IAV NS1 protein suppresses the NLRP3 inflammasome by restraining NF-κB activation and NLRP3 inflammasome [22]. However, the concrete manner of IAV-activated NLRP3 inflammasome, especially in regulating the first step of NLRP3 inflammasome activation remains unknown.

MEK1/2-ERK1/2 signaling pathway is widely known for its essential role in regulating gene expression [27,28]. c-Fos and c-Jun are principal downstream transcriptional factors in the ERK pathway [29]. Activator protein-1 (AP-1), mainly composed of c-Fos and c-Jun, regulates expression levels of pro-inflammatory cytokines [30,31]. However, whether AP-1 is involved in control of NLRP3 inflammasome upon IAV infection requires to be further explored.

Overall, we demonstrate that IAV infection upregulates transcription and maturation of IL-1β. Furthermore, IFN-α/IFN-β suppress NLRP3 inflammasome activation by downregulation of pro-IL-1β mRNA upon IAV infection. Notably, treatment with U0126 and knock-down of c-Jun result in inhibiting IAV infection-mediated NLRP3 inflammasome through downregulating transcription of pro-IL-1β. Thus, we discovered that the AP-1 signaling pathway accelerates activation of NLRP3 inflammasome via upregulating pro-IL-1β mRNA upon IAV infection.

## Materials and methods

### Cells and virus

HEK293T and THP-1 were ordered from China Center of Type Culture Collection (CCTCC) (Wuhan, China). THP-1 were induced by phorbol-12-myristate-13-acetate (PMA, 60 ng/ml) for 12–16 h to differentiate THP-1 macrophages. Influenza A virus H3N2 (A/Hong Kong/498/97) was provided by CCTCC.

### Reagents and antibodies

Polybrene, TRIzol and Lipofectamine 2000 were obtained from Invitrogen (Carlsbad, CA, USA). PMA (Cat#P8139), ATP (Cat#A7699), LPS (Cat#L2630), U0126 (Cat#U120), and Bay11–7082 (Cat#B5556) were obtained from Sigma-Aldrich (St. Louis, MO, USA). Ac-YVAD-cmk (Cat#inh-yvad) was ordered from InvivoGen (San Diego, CA, USA). Pierce^TM^ DSS (Cat#A39267) was ordered from Thermo Scientific (Waltham, MA, USA). IL-1β (human) ELISA Kit II (Cat# 557966) was from BD Biosciences (San Jose, CA, USA). Antibody anti-GAPDH (Cat# 60004-1-lg) was obtained from Proteintech (Wuhan, China). Antibodies anti-NLRP3 (clone ID: D4D8T), IL-1β (clone ID: D3U3E), and Caspase-1 (clone ID: D7F10) were obtained from Cell Signaling Technology (Beverly, MA, USA). Antibodies anti-ASC (clone ID: B-3) and anti-ASC (clone ID: F-9) were obtained from Santa Cruz Biotechnology (Santa Cruz, CA, USA).

### Small interfering RNAs

Small interfering RNAs (siRNA) specific to NF-κB (siR-RelA-p65), AP-1 (siR-c-Jun), and its negative control (siR-NC) were obtained from RiboBio Biotech (Guangzhou, China). Target sequences are listed as followed:

siR-NC: 5'-TTCTCCGAACGTGTCACGT-3;'

siR-p65: 5'-AAGATCAATGGCTACACAGGA-3;'

siR-c-Jun: 5'-CGGACCTTATGGCTACAGTAA-3;'

siR-IL-1β#1: 5'-CGATGCACCTGTACGATCA-3;'

siR-IL-1β#2: 5'-GATGTCTGGTCCATATGAA-3;'

siR-IL-1β#3: 5'-GGATGACTTGTTCTTTGAA-3.'

### Quantitative real-time PCR

Total RNA extraction was extracted using TRIzol reagent and transcribed into complementary DNA (cDNA). We prepared reaction premix by SYBR Quantitative Real-time PCR (qPCR) kits. The mixture contained 10 μl 2 × SYBR Green mix, 1 μl cDNA template, 1 μl specific primers [0.5 μl forward (F) and reverse (R) primer each], and 8 μl ddH_2_O.

All qPCR primers were designed with the available tools online (https://www.ncbi.nlm.nih.gov/). The following primers were used in this study.

**Table:** 

Primer title	Orientation (5'-3')
NLRP3-F/-R	AAGGGCCATGGACTATTTCC**/**GACTCCACCCGATGACAGTT
Caspase-1-F/-R	TCCAATAATGCAAGTCAAGCC**/**GCTGTACCCCAGATTTTGTAGCA
IL-1β-F/-R	CACGATGCACCTGTACGATCA**/**GTTGCTCCATATCCTGTCCCT;
IL-1Ra-F/-R	GATGTGCCTGTCCTGTGTCA**/**ACTCAAAACTGGTGGTGGGG;
ASC-F-/R	AACCCAAGCAAGATGCGGAAG**/**TTAGGGCCTGGAGGAGCAAG;
Rel-F/-R	GATCTTGAGCTCGGCAGTGT**/**CCAGACCAACAACAACCCCT;
c-Jun-F/-R	GCGGACCTTATGGCTACAGT**/**ATCTGTCACGTTCTTGGGGC;
NLRC4-F/-R	ACCTACAGAATCAACGGCTGC**/**ACAGGGTTCACTTGACAGAGAC;
NLRP1-F/-R	CTGCTATCGAAGCCCTGGAG**/**GCTGCTCTCGATACTGGTCC;
IFN-β-F/-R	GACGCCGCATTGACCATCTA**/**TGCTCATGAGTTTTCCCCTGG;
PKR-F/-R	AAAGCGAACAAGGAGTAAG**/**GATGATGCCATCCCGTAG;
OAS1-F/-R	TTCCGTCCATAGGAGCCAC/AAGCCCTACGAAGAATGTC;
Mx1-F/-R	ACCACAGAGGCTCTCAGCAT**/**CTCAGCTGGTCCTGGATCTC;
IL-6-F/-R	GTACATCCTCGACGGCATCTCA**/**GCACAGCTCTGGCTTGTTCCTC;
TNF-ɑ-F/-R	GCTGCACTTTGGAGTGATCG**/**CTTGTCACTCGGGGTTCGAG;
GAPDH-F/-R	AAGGCTGTGGGCAAGG/TGGAGGAGTGGGTGTCG.

### ASC oligomerization assay

THP-1 macrophages were lysed in buffer (50 mM Tris, pH7.5, 150 mM NaCl, 1% NP-40, 5 mM EDTA, and 10% glycerol) at 4°C for 30 min. Lysates were centrifugated at 12,000 rpm for 15 min. The supernatants of the lysates were mixed with 5 × SDS loading buffer for the input sample. The pellets of the lysates were washed with PBS and then cross-linked using DSS (at the final concentration of 2 mM) at 37°C for 30 min. The cross-linked pellets were mixed with 2 × SDS loading buffer for ASC oligomerization analysis.

### Lentiviral production and infection

The experimental procedures of lentiviral production and infection were performed referring to available protocols in the website (http://www.addgene.org/protocols/plko/). The specific sequences of shRNAs were as follows: shNC: 5'-CAACAAGATGAAGAGCACCAA; shNLRP3: 5'-CAGGTTTGACTATCTGTTCT; shASC: 5'−GATGCGGAAGCTCTTCAGTTTCA.

### Statistical analyses

For data with a normal distribution and homogeneity of variance, the difference between two groups was statistically analyzed by a two-tailed Student *t* test. Data are expressed as means ± SD. The evaluation of statistical significance was according to the *P* value. *, *P* < 0.05; **, *P* < 0.01; ***, *P* < 0.001; NS, not-significant, *P* > 0.05.

## Results

### IAV infection induces pro-IL-1β mRNA and secreted IL-1β protein

**Figure 1.**
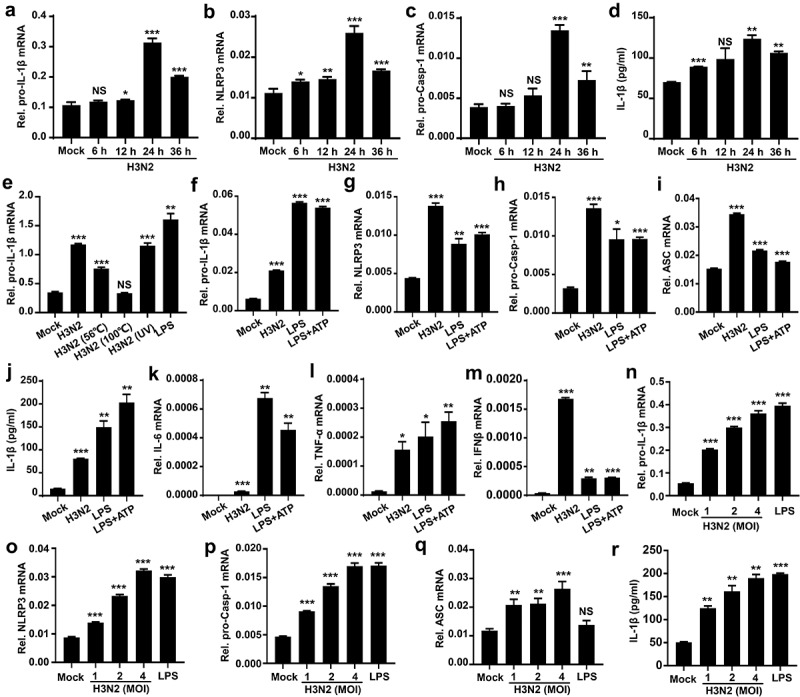
IAV infection induces pro-IL-1β mRNA and secreted IL-1β. (a–d) THP-1 macrophages were infected by H3N2 (MOI = 2) for 6, 12, 24, and 36 h, respectively. the mRNA levels of indicated genes were quantified by qPCR (b and c). Secreted IL-1β were detected by ELISA (d). (e) THP-1 macrophages were treated with live H3N2, heated-inactivated H3N2, or UV-inactivated H3N2 at an MOI of 2 for 24 h, accompanied with LPS (1 μg/ml) for 6 h or ATP (5 mM) for 30 min as positive controls. (f–m) THP-1 macrophages were infected by H3N2, accompanied with LPS (1 μg/ml) for 6 h or ATP (5 mM) for 30 min, respectively. the mRNA levels of the indicated genes were quantified by qPCR (f-m). Secreted IL-1β was analyzed by ELISA (J). (N–R) H3N2 (MOI = 1, 2, or 4) infected THP-1 macrophages for 24 h, with the LPS (1 μg/ml, positive control) stimulation for 6 h. the mRNA levels of the indicated genes were analyzed by qPCR (n-q). Secreted IL-1β were analyzed by ELISA (r).

IL-1β plays essential functions in immune response and regulation of downstream pro-inflammatory cytokines [32]. To investigate the potential action of immune response to IAV infection, THP-1 macrophages were first infected with IAV strain (H3N2). Our results suggested that H3N2 infection significantly accelerates pro-IL-1β (Figure 1(a)), NLRP3 (Figure 1(b)), and pro-Capsase-1 (Figure 1(c)) transcription, and also enhanced secreted IL-1β (Figure 1(d)) in time-dependent fashions. Notably, pro-IL-1β mRNA was significantly induced by H3N2, the inoculation of ultraviolet (UV)-inactivated H3N2, and the treatment of Lipopolysaccharides (LPS) (a positive control), whereas the pro-IL-1β mRNA was unaffected by inoculation of heat-inactivated H3N2 (Figure 1(e)), suggesting the viral RNA is closely related to regulating pro-IL-1β transcription.

To explore the function of IAV infection on NLRP3 inflammasome, the cells were stimulated by H3N2, LPS, and LPS+ATP (positive control), respectively. We noted that pro-IL-1β (Figure 1(f)), NLRP3 (Figure 1(g)), pro-caspase-1 (Figure 1(h)), ASC (Figure 1(i)) mRNAs, and secreted IL-1β protein (Figure 1(j)) were significantly induced in H3N2-infected, LPS-treated, and LPS+ATP-stimulated cells. We also noted that the levels of cytokines, including IL-6 (Figure 1(k)), TNF-α (Figure 1(l)), and IFN-β (Figure 1(m)) mRNAs were induced in H3N2-infected, LPS-treated, or LPS+ATP-stimulated cells, respectively. Moreover, when H3N2 infected THP-1 macrophages at different doses or LPS stimulates the cells, pro-IL-1β (Figure 1(n)), NLRP3 (Figure 1(o)), pro-Caspase-1 (Figure 1(p)), and ASC (Figure 1(q)) mRNAs, and secreted IL-1β protein (Figure 1(r)) were significantly induced upon H3N2 infection in dose-dependent manners. Altogether, these data suggest that H3N2 infection promotes pro-IL-1β and NLRP3 transcription, thereby triggering inflammatory responses upon IAV infection.

### IAV infection promotes NLRP3 inflammasome activation

**Figure 2.**
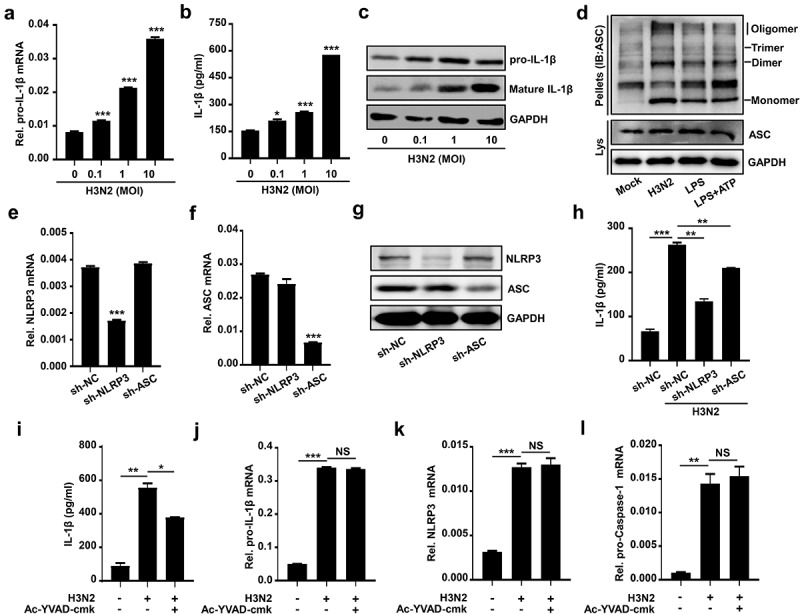
IAV infection promotes NLRP3 inflammasome activation. (a–c) H3N2 (MOI = 0, 0.1, 1.0, and 10) infected THP-1 macrophages for 24 h. the pro-IL-1β mRNA level was analyzed by qPCR (a). Secreted IL-1β was detected by ELISA (b). the precursor and maturation of IL-1β, coupled with inter control GAPDH, were analyzed by using indicated antibodies by Western blotting (c). (d) THP-1 macrophages were treated with live H3N2 (MOI = 2), LPS (1 μg/ml) for 6 h, or LPS (1 μg/ml) for 6 h with ATP (5 mM) for 30 min, respectively. Lysates and ASC oligomerization were detected by indicated antibodies. (e–g) NLRP3 (e) and ASC (f) mRnas were assured by qPCR and NLRP3 (g), while ASC (g) proteins were detected by Western blotting in macrophages infected by lentiviruses expressing the indicated shRNA. (h) H3N2 (MOI = 2) infected macrophages stably expressing shRNA against target genes. IL-1β protein were detected by ELISA. (i–l) H3N2 (MOI = 2) infected macrophages for 24 h, and then the cells were treated with Ac-YVAD-cmk (1 mm) for 6 h. Secreted IL-1β was detected by ELISA (i). the mRNA levels of the indicated genes (i–k) were analyzed by qPCR.

To investigate whether secreted IL-1β was involved in H3N2-activated NLRP3 inflammasome, H3N2 infected THP-1 macrophages at different doses, pro-IL-1β mRNA (Figure 2(a)), secreted IL-1β protein (Figure 2(b)), and intracellular precursor and maturation of IL-1β protein (Figure 2(c)) were increased upon H3N2 infection. We also found ASC oligomerization was significantly enhanced upon H3N2 infection, similar to the ASC oligomerization induced by LPS or LPS + ATP stimulation as expected (Figure 2(d)). To further investigate whether IAV promotes secreted IL-1β *via* activating NLRP3 inflammasome activation, we constructed short hairpin RNAs (shRNAs) and generated THP-1 stably expressed shNLRP3 or shASC. The knock-down efficiency was identified through detecting NLRP3 (Figure 2(e)) and ASC (Figure 2(f)) mRNA and corresponding proteins (Figure 2(g)) expression. In the THP-1 cell stably expressed the indicated shRNAs infected by IAV, the secreted IL-1β was increased upon IAV infection, whereas this increment was obviously weakened by shNLRP3 or shASC, respectively (Figure 2(h)).

The detailed mechanism involved in the secreted IL-1β mediated by H3N2 was further investigated. Ac-YVAD-cmk was used to suppress the activity of mature Caspase-1. Our results revealed that upregulation of IAV-induced secreted IL-1β was significantly reduced by Ac-YVAD-cmk (Figure 2(i)), whereas upregulation of pro-IL-1β (Figure 2(j)), NLRP3 (Figure 2(k)), and pro-Caspase-1 (Figure 2(l)) mRNAs mediated by H3N2 was not influenced by Ac-YVAD-cmk. Overall, these results illustrated that IAV infection activates NLRP3 inflammasome companied with the upregulation of pro-IL-1β transcription.

### IAV-Mediated NLRP3 inflammasome activation is primed by the IAV-induced pro-IL-1β mRNA level

**Figure 3.**
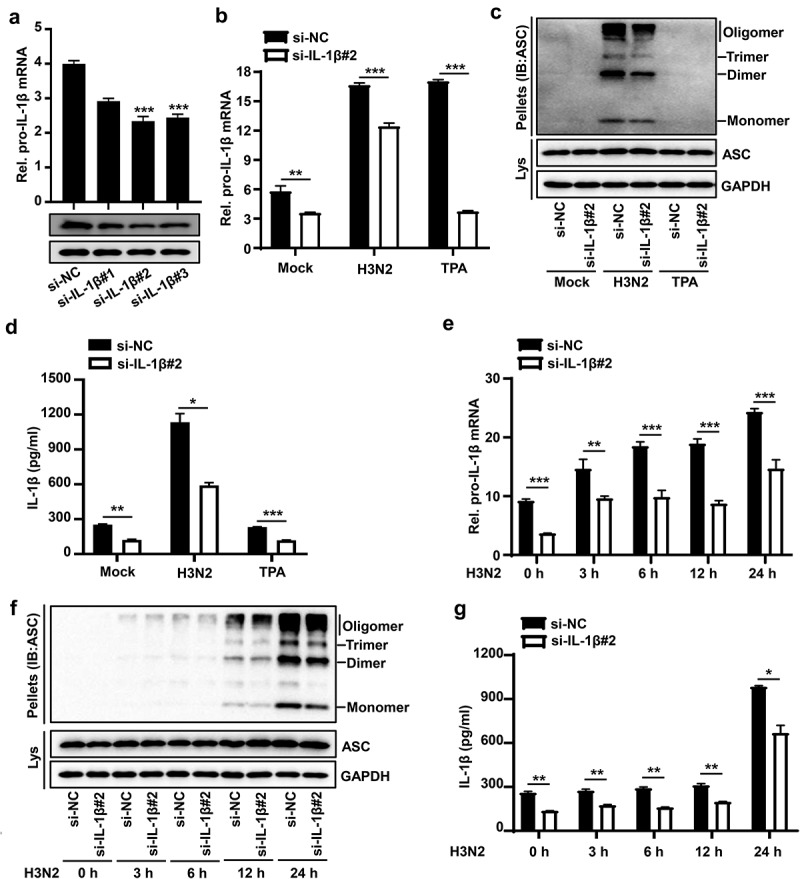
IAV-Induced pro-IL-1β mRNA facilitates NLRP3 inflammasome activation. (a) A certain amount (50 nM) of si-NC (negative control), si-IL-1β#1, si-IL-1β#2, and si-IL-1β#3 were delivered into THP-1 macrophages by Lipofectamine 2000 for 36 h. the pro-IL-1β mRNA level was analyzed by qPCR and pro-IL-1β protein was detected by Western blotting. (b–d) the transfected macrophages were infected with H3N2 (MOI = 4) for another 24 h or were treated with TPA (50 ng/ml) for 6 h. the mRNA levels of pro-IL-1β (b) were quantified by qPCR. Lysates and ASC oligomerization were detected by the indicated antibodies (c). Secreted IL-1β was analyzed by ELISA assay (d). (e–g) A certain amount (50 nM) of si-IL-1β#2 or si-NC were delivered into macrophages, and then the cells were infected with H3N2 (MOI = 4) for different periods (0 h, 3 h, 6 h, 12 h, or 24 h). the mRNA levels of pro-IL-1β (e) were analyzed by qPCR. Lysates and ASC oligomerization were detected by the indicated antibodies (f). Secreted IL-1β was detected by ELISA (G).

To investigate whether IAV-induced pro-IL-1β mRNA was involved in activation of NLRP3 inflammasome. The efficiency of siRNA against IL-1β (si-IL-1β) was examined in THP-1 macrophages, revealing that si-IL-1β#2 was robustly effective against IL-1β in THP-1 macrophages (Figure 3(a)). To further investigate whether IAV-induced pro-IL-1β mRNA affect NLRP3 inflammasome activation, si-IL-1β#2 or si-NC (Negative control) were delivered into THP-1 macrophages, and then the cells were treated with H3N2 infection or TPA. The knock-down efficiency of si-IL-1β-2 was identified through detecting the mRNA level of pro-IL-1β (Figure 3(b)). The results indicated that IAV-induced pro-IL-1β mRNA facilitates IAV-mediated NLRP3 inflammasome activation by detecting ASC oligomerization (Figure 3(c)) and secreted IL-1β (Figure 3(d)), whereas the boosted pro-IL-1β mRNA induced by TPA failed to activate NLRP3 inflammasome (Figure 3(c,d)).

To further investigate the relationship between IAV-induced pro-IL-1β mRNA and IAV-mediated NLRP3 inflammasome activation, si-IL-1β#2 or si-NC were delivered into THP-1 macrophages, and then the cells were treated with H3N2 at different time points. The knock-down efficiency of si-IL-1β#2 was identified through detecting mRNA level of pro-IL-1β (Figure 3(e)). The results indicated that IAV-induced pro-IL-1β mRNA occurred prior to IAV-mediated NLRP3 inflammasome activation (Figure 3(e–g)), and IAV-induced pro-IL-1β mRNA indeed facilitated IAV-mediated NLRP3 inflammasome activation by ASC oligomerization (Figure 3(e)) and resulted in the secretion of IL-1β protein (Figure 3(g)). Thus, IAV-induced pro-IL-1β mRNA facilitates IAV-mediated NLRP3 inflammasome activation.

### Type I IFN inhibits IAV-activated NLRP3 inflammasome by downregulating pro-IL-1β mRNA

**Figure 4.**
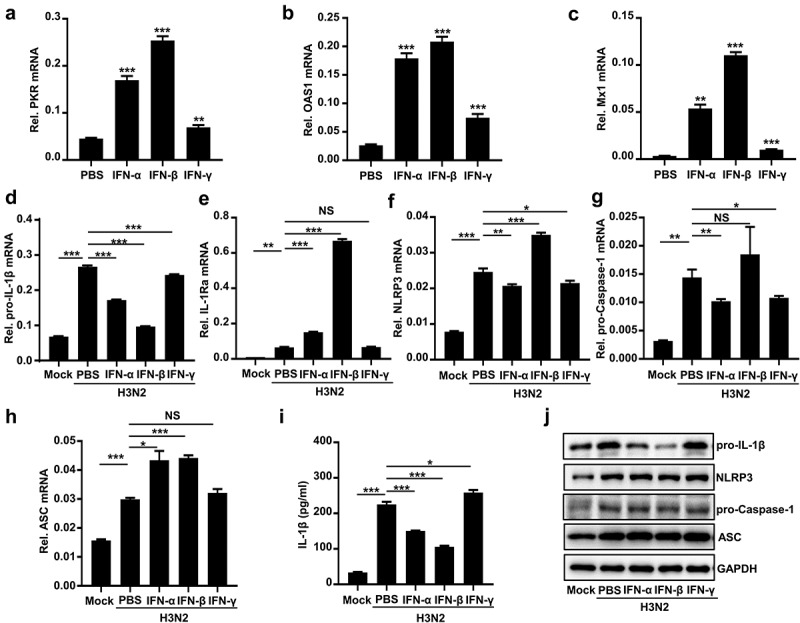
Type I IFN inhibits IAV-activated NLRP3 inflammasome by downregulating pro-IL-1β mRNA. (a–c) IFN-α (600 U/ml), IFN-β (50 ng/ml), and IFN-γ (50 ng/ml) stimulated THP-1 macrophages for 6 h. the PKR (a), OAS1 (b) and M x 1 (c) mRnas were quantified by qPCR. (d–j) H3N2 (MOI = 2) infected macrophages for 24 h, and then the cells were treated with IFN-α protein (600 U/ml), IFN-β (50 ng/ml), or IFN-γ (50 ng/ml) protein for 6 h. the mRNA levels of the indicated genes (d-h) were analyzed by qPCR. Secreted IL-1β was detected by ELISA (i). the levels of indicated proteins were detected by Western blotting (j).

Type I IFN (IFN-α/β) has been found to suppress NLRP3 inflammasome activation by two different mechanisms [33]. First, the IFN signaling represses the NLRP3 inflammasome activity *via* STAT1 to suppress secreted IL-1β. Second, IFN-α/β upregulates IL-10 production to reduce abundances of pro-IL-1β *via* STAT3. Here, we further evaluated the regulatory functions of IFN-α/β/γ on the secreted IL-1β. The mRNAs of interferon-stimulated genes (ISGs), including PKR (Figure 4(a)), OAS1 (Figure 4(b)), and M × 1 (Figure 4(c)), were induced by IFN-α/β/γ proteins in THP-1 macrophages, confirming normal actions of the IFNs.

THP-1 macrophages were infected by H3N2 and then stimulated with PBS, IFN-α, IFN-β, and IFN-γ proteins, respectively. IFN-α/β reduced H3N2-induced pro-IL-1β mRNA (Figure 4(d)), whereas promoted H3N2-induced IL-1Ra (Interleukin 1 receptor antagonist) mRNA (Figure 4(e)). However, IFN-γ had no significant effect on these changes (Figure 4(d,e)). Interestingly, we noted that IFN-α, IFN-β, and IFN-γ proteins displayed no significant effects on the mRNA of NLRP3 (Figure 4(f))(Figure 4(F)),^1^ pro-caspase-1 (Figure 4(g)), and ASC (Figure 4(h)) induced by H3N2 infection. Notably, IFN-α and IFN-β inhibited pro-IL-1β proteins and secreted IL-1β protein induced by H3N2 infection (Figure 4(i,j)), while IFN-γ had no similar effects (Figure 4(i,j)). Altogether, our results suggested that IFN-α/β suppresses activation of NLRP3 inflammasome *via* attenuating pro-IL-1β mRNA upon H3N2 infection.

### AP-1 signaling inhibitor U0126 specifically inhibits IAV-induced NLRP3 inflammasome activation by downregulating pro-IL-1β mRNA

**Figure 5.**
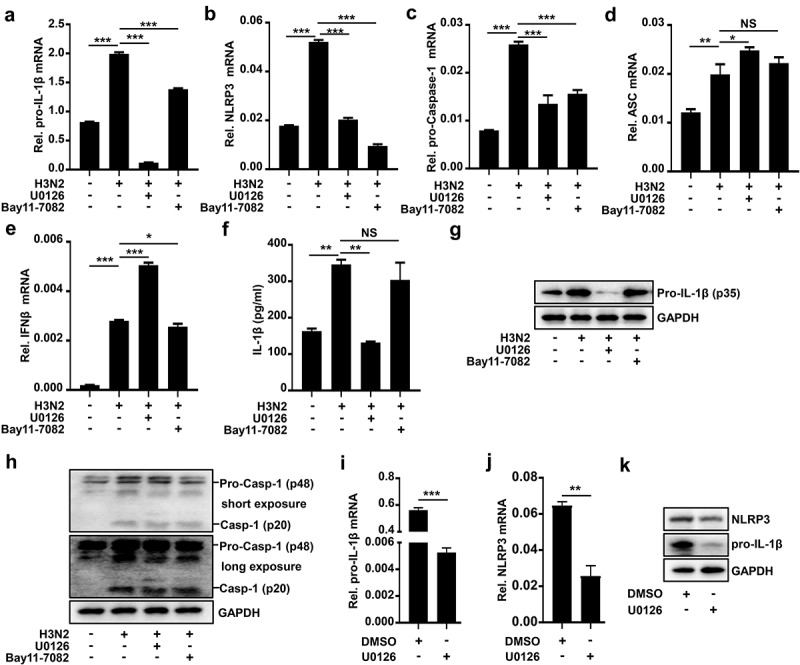
U0126 specifically inhibits IAV-induced NLRP3 inflammasome activation by downregulating pro-IL-1β mRNA. (a–h) H3N2 (MOI = 2) infected THP-1 macrophages for 24 h, and then the cells were stimulated with U0126 (10 μM) or Bay11–7082 (20 μM) for 6 h. the mRNA levels of the indicated genes (a–e) were analyzed by qPCR. Secreted IL-1β protein was detected by ELISA assay (f). Pro-IL-1β and GAPDH proteins were detected by Western blotting (g). Pro-Caspase-1, mature Caspase-1, and GAPDH proteins were detected by Western blotting (h). THP-1 macrophages were treated with DMSO or U0126 (10 μM). the mRNA levels of the indicated genes (i–j) were analyzed by qPCR, while the indicated protein levels were analyzed by Western blotting (k).

Next, how H3N2 infection regulated pro-IL-1β transcription was investigated. The macrophages were infected by H3N2 and subsequently treated with DMSO, U0126 (a selective inhibitor of MEK-1 and MEK-2), and Bay-11–7082 (an NF-κB inhibitor), respectively. Notably, pro-IL-1β mRNA was boosted upon H3N2 infection, H3N2-induced transcription of pro-IL-1β was inhibited by U0126 and mildly attenuated by Bay-11–7082 (Figure 5(a)). Importantly, both U0126 and Bay-11–7082 significantly repressed H3N2-induced NLRP3 mRNA (Figure 5(b)) and pro-Caspase-1 mRNA (Figure 5(c)), but had no significant effect on H3N2-induced ASC mRNA (Figure 5(d)) and IFN-β mRNA (Figure 5(e)). Interestingly, we noted that secreted IL-1β induced by H3N2 was significantly repressed by U0126, but not by Bay-11–7082 (Figure 5(f)), suggesting that AP-1 signaling pathway is important for IL-1β protein secretion upon IAV infection. Importantly, pro-IL-1β protein induced by H3N2 was significantly repressed by U0126 (Figure 5(g)). We noted that mature Caspase-1 protein induced by H3N2 was slightly repressed by U0126 (Figure 5(h)). The results are consistent with IAV-induced pro-IL-1β mRNA facilitates IAV-mediated NLRP3 inflammasome activation.

To further explore function of U0126, macrophages were treated with DMSO or U0126, pro-IL-1β transcription was markedly inhibited by U0126 (Figure 5(i)), while NLRP3 transcription was mildly attenuated by U0126 (Figure 5(j)). Meanwhile, the protein expression of pro-IL-1β was also markedly inhibited by U0126, and NLRP3 protein was mildly attenuated by U0126 (Figure 5(k)). These results suggested that U0126 repressed IAV-mediated secreted IL-1β protein *via* mainly downregulating pro-IL-1β mRNA. It’s well known that NF-κB is critical for pro-IL-1β transcription in inflammasome activation [2], which is consistent with our results of the inhibition of Bay-11–7082 on production of pro-IL-1β mRNA. Unlike Bay-11–7082, U0126 displayed a comparative superiority on the suppression of transcription and maturation of IL-1β, indicating that AP-1 signaling pathway played a dominant role in transcription and maturation of IL-1β induced by H3N2.

### Knock-Down of c-Jun prohibits IAV-activated NLRP3 inflammasome by downregulating pro-IL-1β mRNA

**Figure 6.**
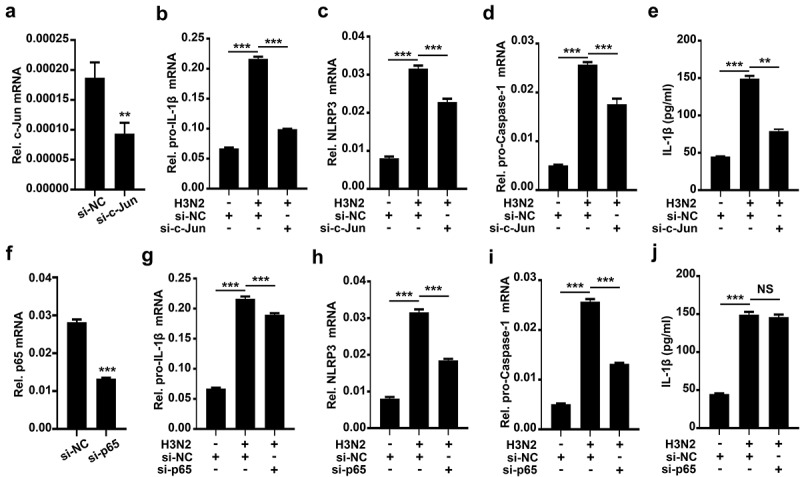
Knock-Down of c-Jun prohibits IAV-activated NLRP3 inflammasome by downregulating pro-IL-1β mRNA. (a) A certain amount (50 nM) of si-c-Jun (against c-Jun) or si-NC (negative control) were delivered into THP-1 macrophages by Lipofectamine 2000. the c-Jun mRNA level was analyzed by qPCR. (b–e) A certain amount (50 nM) si-c-Jun or si-NC were delivered into macrophages for 12 h, and then the cells were infected with H3N2 (MOI = 2) for another 24 h. the mRNA levels of the indicated genes (b-d) were quantified by qPCR. Secreted IL-1β was analyzed by ELISA (e). (f) A certain amount (50 nM) si-p65 (against p65) or si-NC were delivered into macrophages. the mRNA of p65 were analyzed by qPCR. (g–j) A certain amount (50 nM) si-p65 or si-NC were delivered into macrophages for 12 h, and then infected with H3N2 (MOI = 2) for another 24 h. the mRNA levels of the indicated gene (f-i) were analyzed by qPCR. Secreted IL-1β was detected by ELISA (j).

How AP-1 signaling pathway and NF-kB signaling pathway activate NLRP3 inflammasome upon IAV infection was further elucidated. Initially, we identified the efficiency of si-c-Jun (siRNA against c-Jun, the subunit of AP-1) in THP-1 macrophages (Figure 6(a)). H3N2-induced pro-IL-1β (Figure 6(b)), NLRP3 (Figure 6(c)), pro-Caspase-1 (Figure 6(d)) mRNAs, and secreted IL-1β protein (Figure 6(e)) were significantly decreased in the presence of si-c-Jun, suggesting that AP-1 plays an essential position in transcription and maturation of IL-1β during H3N2 infection.

**Figure 7.**
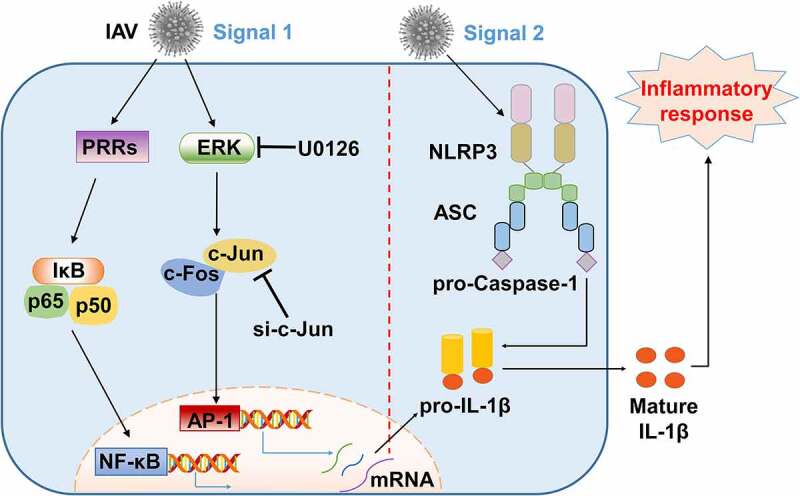
A proposed mechanism by which AP-1 signaling activates NLRP3 inflammasome upon IAV infection. Two intracellular signaling pathways, AP-1 and NF-κB, are involved in the occurrence of the downstream events through two types of mechanisms upon IAV infection. In in^2^ the first signal, activation of NLRP3 inflammasome is primed by transcription of pro-IL-1β through ERK/c-Jun/AP-1 signaling pathway, and IL-1β protein is subsequently accumulated upon IAV infection. In in2 the second signal, IAV infection activates the NLRP3 inflammasome by facilitating the binding of ASC to NLRP3 and providing a platform for pro-Caspase-1 activation, allowing the maturation and secretion of IL-1β to undergo inflammatory responses.

We also identified the efficiency of si-p65 (siRNA against p65, the subunit of NF-κB) in THP-1 macrophages (Figure 6(f)). Knock-down of p65 modestly inhibited pro-IL-1β mRNA expression (Figure 6(g)), while markedly inhibited transcription of NLRP3 (Figure 6(h)) and pro-Caspase-1 (Figure 6(i)), whereas had no effects on secreted IL-1β production (Figure 6(j)) induced by H3N2, suggesting NF-κB pathway behaviors an inconspicuous regulation on H3N2-activated NLRP3 inflammasome. Thus, these results demonstrated that c-Jun plays important roles in transcription and maturation of IL-1β, and further indicated that AP-1 signaling pathway acts as essential functions in NLRP3 inflammasome activation (Figure 7).

## Discussion

NLRP3 inflammasome regulates immune response against several RNA viruses, such as IAV and Enterovirus 71 (EV71) [21,34–36]. Some RNA viruses activate NLRP3 inflammasome through first signal and second signals, such as VSV and encephalomyocarditis virus (EMCV) [37]. IAV activated NLRP3 inflammasome in different fashions, including the first step and second step [22,38–43].

AP-1, the main substrate of the multiple MAPK pathways, is stimulated by a series of stimuli, including stress and cytokines [44]. Interestingly, C3G4 (cyanidin-3-glucoside) activated NLRP3 inflammasome through AP-1 signaling pathway in the epithelial cells [45]. PRRSV enhances secreted IL-1β by AP-1 signaling pathway in microglia [46]. Our findings presented in this study show that AP-1 contributes to H3N2-mediated NLRP3 inflammasome activation, which extends functional scope of AP-1 in the regulation of NLRP3 inflammasome.

Previous study suggested that NF-κB is essential to accelerate the transcription of pro-IL-1β and NLRP3 [2]. It was also reported that the MyD88/ERK/AP-1 signaling pathway promotes IL-1β production by upregulating pro-IL-1β mRNA, while an inhibitor of NF-κB, BAY11–7082, had no significant influence on promotion of secreted IL-1β during PRRSV infection [46]. Inversely, NF-κB signaling and MAPK signaling promote the NLRP3 inflammasome activation during ischemic stroke in neurons [47]. Our results uncovered that NF-κB is critical for upregulating NLRP3 and pro-Caspase-1 mRNAs, whereas AP-1 is indeed important for upregulating mRNA levels of pro-IL-1β, NLRP3, and pro-Caspase-1. Notably, compared with NF-κB signaling pathway, AP-1 functions a prominent position in upregulating IL-1β transcription and maturation upon IAV infection. These findings uncovered that the mRNA levels of pro-IL-1β, NLRP3, or pro-Caspase-1 were regulated by different signaling pathways, especially the different types of stimuli.

Type I IFN significantly eliminates NLRP3 inflammasome by especially downregulating pro-IL-1β mRNA, which may control the threshold of NLRP3 inflammasome-modulated IL-1β production upon IAV infection. Our results demonstrate that AP-1 plays a superior position in NLRP3 inflammasome-mediated IL-1β secretion upon IAV infection relative to NF-κB. We assumed that AP-1 promotes NLRP3 inflammasome-mediated IL-1β activation by targeting the first signal during RNA virus infection, although the assumption still requires it to be further explored.

In short, we demonstrate that AP-1 plays an essential role in H3N2-activated NLRP3 inflammasome by upregulating pro-IL-1β mRNA transcription. Furthermore, AP-1 signaling pathway may provide a potential target for therapeutic interventions on IAV-caused inflammatory diseases.

## Data Availability

The authors confirm that the data supporting the findings of this study are available within the article and/or its supplementary materials.

## Endnotes

1. The spelling of ”Figure 4(F)” is wrong. ”Figure 4(F)” is replaced by ”Figure 4(f)”

2. The spelling of ”in” is wrong. ”in” is replaced with ”In”.
